# The impact of different thresholds on optical coherence tomography angiography images binarization and quantitative metrics

**DOI:** 10.1038/s41598-021-94333-y

**Published:** 2021-07-20

**Authors:** Alessandro Arrigo, Emanuela Aragona, Andrea Saladino, Alessia Amato, Francesco Bandello, Maurizio Battaglia Parodi

**Affiliations:** Department of Ophthalmology, IRCCS San Raffaele Scientific Institute, Vita-Salute University, via Olgettina 60, 20132 Milan, Italy

**Keywords:** Biomarkers, Medical research

## Abstract

Optical coherence tomography angiography (OCTA) provides several data regarding the status of retinal capillaries. This information can be further enlarged by employing quantitative metrics, such as vessel density (VD). A mandatory preliminary step of OCTA quantification is image binarization, a procedure used to highlight retinal capillaries on empty background. Although several binarization thresholds exist, no consensus is reached about the thresholding technique to be used. In this study, we tested thirteen binarization thresholds on a dataset made by thirty volunteers. The aim was to assess the impact of binarization techniques on: (I) detection of retinal capillaries, assessed by the calculation of overlapping percentages between binarized and original images; (II) quantitative OCTA metrics, including VD, vessel tortuosity (VT) and vessel dispersion (Vdisp); (III) foveal avascular zone (FAZ) detection. Our findings showed Huang, Li, Mean and Percentile as highly reliable binarization thresholds (p < 0.05), whereas the worst binarization thresholds were Intermodes, MaxEntropy, RenylEntropy and Yen (p < 0.05). All the thresholds variably underestimated VD metric and FAZ detection, with respect to the original OCTA images, whereas VT and Vdisp turned out to be more stable. The usage of a Fixed threshold resulted extremely useful to reduce VD and FAZ underestimations, although bound to operators’ experience.

## Introduction

Optical coherence tomography angiography (OCTA) is a largely used, non-invasive technique providing extremely detailed information regarding intraretinal vessels and choriocapillaris^1^. The most used quantitative method to analyze OCTA data is vessel density (VD), intended as the ratio between vessels’ signal and empty spaces^2^. More recently, new quantitative OCTA parameters have been introduced, trying to provide more detailed information about the perfusion status of the retina in healthy and pathological conditions^3–5^. On the other side, another largely used parameter is represented by foveal avascular zone (FAZ) area, which is a poorly specific but highly sensitive biomarker of intraretinal perfusion impairment^6^. FAZ can be manually or automatically segmented, and it is conventionally excluded from VD calculation. Worthily, the most of these metrics require a preliminary step, represented by image binarization. This procedure transforms OCTA greyscale reconstructions into black-white images, thus highlighting the vessels in the context of the empty background. Although it is known that the choice of the binarization threshold may strongly influence the reliability of the final images, to date, no guideline is available regarding the proper binarization threshold to be applied and no consensus has been reached about the reliability estimate of this procedure^7,8^. The main aim of the present study is to investigate the impact of the main thresholding techniques on a cohort of healthy eyes analyzed by OCTA and to assess the influence of quantitative OCTA metrics.

## Materials and methods

The study was designed as experimental, cross-sectional investigation. Healthy volunteers with no ocular or systemic diseases were recruited at the Ophthalmology Unit of IRCCS San Raffaele Scientific Institute, Milan, Italy. All the volunteers signed an informed consent before the inclusion. The study was approved by the ethical committee of IRCCS San Raffaele Scientific Institute (MIRD2020) and it was conducted in accordance with Helsinki declaration.

We included only one eye from each patient, randomly selected. A complete ophthalmologic examination was performed to confirm the absence of any ocular or systemic disease, or any kind of media opacity.

OCTA 3 × 3 mm high-resolution images were acquired by means of a Swept source DRI Topcon Triton device (Topcon Inc., Japan). Topical administration of Phenylephrine was used for all the eyes 20 min before OCTA acquisitions. Furthermore, 0.2% sodium hyaluronate artificial drop was instilled immediately before OCTA acquisition. Images’ quality was assessed by Topcon Quality Index (TQI), and we included only reconstructions with TQI > 70. Superficial capillary plexus (SCP), deep capillary plexus (DCP) and choriocapillaris (CC) were automatically segmented by ImageNet6 software. An expert grader (AA) checked all the segmentations slabs, which were eventually manually corrected. Moreover, the same grader carefully checked the presence of OCTA artifacts, including motion, blinking and projections artifacts^1^. All the reconstructions showing OCTA artifacts were excluded, and the interested eyes were eventually reacquired.

We planned three different experiments. The first was focused in achieving reference OCTA images to be used to compare the results of all the other binarization thresholds. The second experiment was conducted to evaluate quantitative OCTA parameters, for each binarized image. The third experiment was focused on the assessment of FAZ detection on each binarized image.

All the experiments were conducted by uploading the images in Fiji software toolbox^9^. Since “Mean” threshold, intended as the mean of all the values of each OCTA reconstruction, is currently the most used one, we included also this binarization threshold as reference. Since most of the evaluations were performed by two independent expert graders, inter-graders correlation coefficient (ICC) was calculated to evaluate the agreement between the two operators. For all the experiments, we compared the images by using the following pipeline: Process → Image Calculator → Subtract (original image – binarized image). The result of the subtraction was then used to calculate the percentage of overlapping between the binarized image and the original image. We arbitrarily considered the following percentages of overlapping: < 20%, < 80% and > 80%.

**Figure 1:**
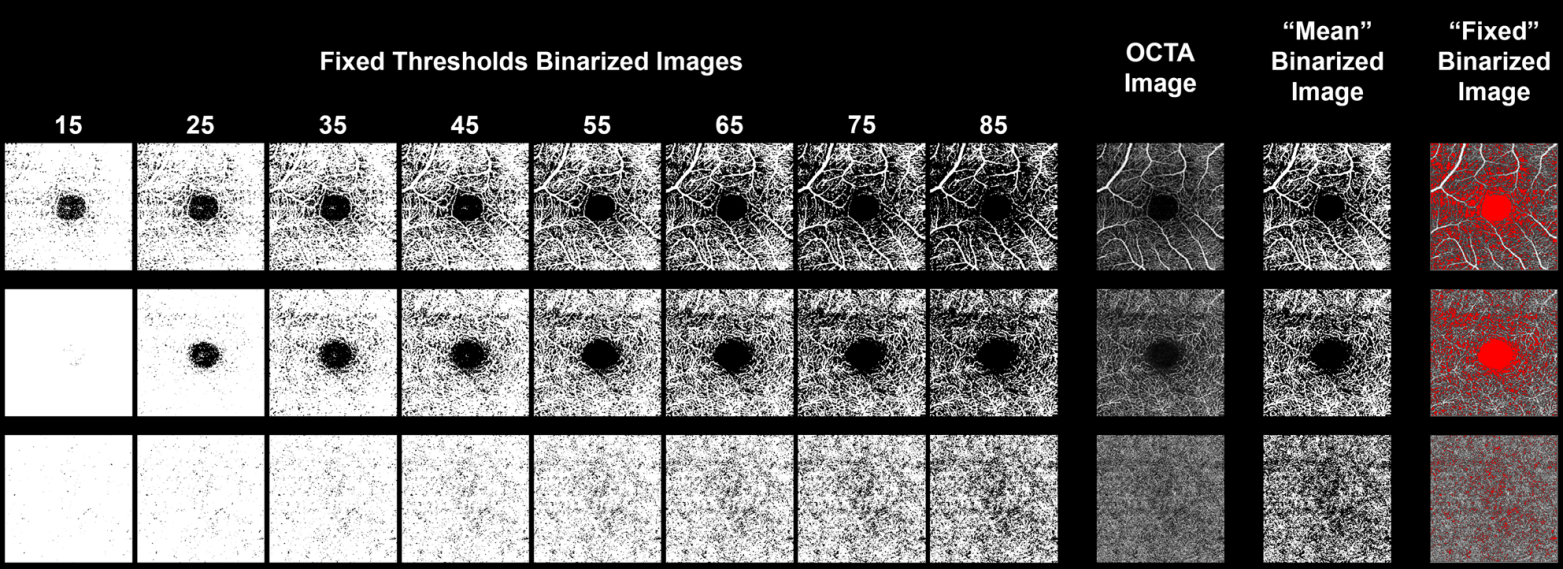
Fixed thresholding tests. SCP, DCP and CC reconstructions are respectively shown in upper, middle and lower lines. Original OCTA reconstructions and “Mean” threshold binarized images are shown as well. The fixed values chosen are 65 for SCP and DCP, and 85 for CC, resulting in the most reliable selection of capillaries, if compared to the original OCTA reconstructions, with respect to the empty background (highlighted in red).

The rationale of the first experiment was to obtain binarized reference images, showing the best matching with the non-binarized original OCTA reconstructions, to be used for the other two experiments. Considering the grayscale distribution of the images, included between 0–255, we performed subsequent tests with increasing fixed thresholds (Fig. 1). Then, two expert graders (AA, EA) checked the effect of each fixed value on the proper inclusion of intraretinal capillaries, by selecting the best fixed threshold value, compared to the original OCTA images, by using the above-described pipeline.

The second experiment was focused on the assessment of the effect of binarization thresholds on quantitative OCTA metrics. We tested 13 different binarization thresholds, available in Fiji toolbox, namely: Default^10^, Huang^11^, Intermodes^12^, Li^13^, Max Entropy^14^, Mean^15^, Moments^16^, Otsu^17^, Percentile^18^, Renyl Entropy^14^, Shanbhag^19^, Yen^20^, and the fixed threshold established by the first experiment. Default thresholding is one of the simplest way to binarize images, resulting from (average intensity background + average intensity object)/2)^10^. Huang thresholding includes two steps: (I) recognition of the objects and the background grayscale levels; (II) adoption of Shannon's entropy function to minimize the fuzziness of the image^11^. Intermodes thresholding is based on the iterative smoothing of a histogram using a running average of size 3, until the recognition of two local maxima: j and k, which are used to compute the final threshold on the basis of (j + k)/2^12^. Li thresholding uses an iterative method to obtain the threshold that minimizes the minimum cross entropy of the original image and its segmented version^13^. Max Entropy thresholding is a technique based on a priori maximation of the entropy of a grayscale histogram^14^. Mean thresholding adopts the mean value of the grayscale level of each image to compute the final threshold^15^. Moments thresholding is based on the moment-preserving principle, namely the deterministic computation of the threshold values in order to preserve the moments of the input image in the output image^16^. Otsu thresholding is a clustering algorithm including the calculation of the weighted sum of variances of two classes of images, then the computation of a threshold value minimizing the intra-class variance^17^. Percentile thresholding calculates the distribution of the grayscale levels and then assumes that the fraction of foreground pixels is 0.5^18^. Renyl Entropy thresholding is a similar procedure of Max Entropy thresholding, but it adopts Renyi's entropy approach to quantify the entropy of the grayscale histogram^14^. Shanbhag thresholding is another entropy-based approach, calculating the total entropy of each grayscale histogram and finding the threshold value maximizing it^19^. Similarly, Yen thresholding is a further grayscale histogram entropy-based calculation approach^20^.

The binarized images were quantitatively inspected by the two independent graders, comparing the binarized images with the original OCTA reconstructions by calculating the percentage of overlapping between binarized and original image, and attributing the following Quality Score: 0 (< 20% of overlapping), 1 (< 80% of overlapping), 2 (> 80% of overlapping). All the binarized images were used for the calculation of VD, vessel tortuosity (VT) and vessel dispersion (VDisp) OCTA quantitative parameters. These metrics were calculated by in-house scripts, in the same ways described in our previous studies^3–5^. The resulting values were statistically analyzed by means of Student T-test (SPSS software Package, Chicago, Illinois, USA). We set statistical significance to p < 0.05.

The same binarization thresholds used in the second experiment were tested focusing on the detection of the FAZ. Also in this case, the two independent graders quantitatively categorized images accordingly to the percentage of overlapping: < 20% of overlapping, < 80% of overlapping and > 80% of overlapping. Then, they calculated the number of eyes matching with these three categories.

## Results

We included 30 eyes of 30 healthy volunteers (15 males; mean age 35 ± 6). The ophthalmologic examination reported no ocular or systemic diseases, clear media and best corrected visual acuity of 0.0 ± 0.0 (20/20 Snellen equivalent) for all the eyes.

The testing of the fixed binarization threshold showed the following values as the best for SCP and DCP, as highlighted by the higher agreement between readers: 65 (ICC 0.89 for SCP and 0.85 for DCP) and 75 (ICC 0.75 for SCP and 0.79 for DCP). For CC, the best values turned out to be 75 (ICC 0.84) and 85 (ICC 0.88). Based on these findings, we considered as fixed thresholding values 65 for SCP and DCP, and 85 for CC. The measurement of VD showed different values accordingly to the fixed threshold adopted. Considering the chosen fixed thresholding values, VD turned out to be significantly higher than the values obtained after “Mean” thresholding, for all vascular plexa (all p < 0.05) (Table 1).

**Table 1:** Vessel density values depending on the fixed threshold adopted and comparison with “Mean” threshold.

Threshold	SCP	DCP	CC
Fixed 15	0.94 ± 0.01	0.97 ± 0.01	0.99 ± 0.01
Fixed 25	0.87 ± 0.02	0.95 ± 0.02	0.99 ± 0.01
Fixed 35	0.77 ± 0.01	0.87 ± 0.02	0.97 ± 0.01
Fixed 45	0.67 ± 0.02	0.76 ± 0.01	0.95 ± 0.01
Fixed 55	0.56 ± 0.01	0.66 ± 0.02	0.89 ± 0.01
Fixed 65	0.46 ± 0.01	0.57 ± 0.02	0.72 ± 0.01
Fixed 75	0.37 ± 0.01	0.48 ± 0.02	0.70 ± 0.01
Fixed 85	0.29 ± 0.01	0.40 ± 0.02	0.69 ± 0.01
Mean	0.42 ± 0.01	0.44 ± 0.02	0.50 ± 0.01

**Figure 2:**
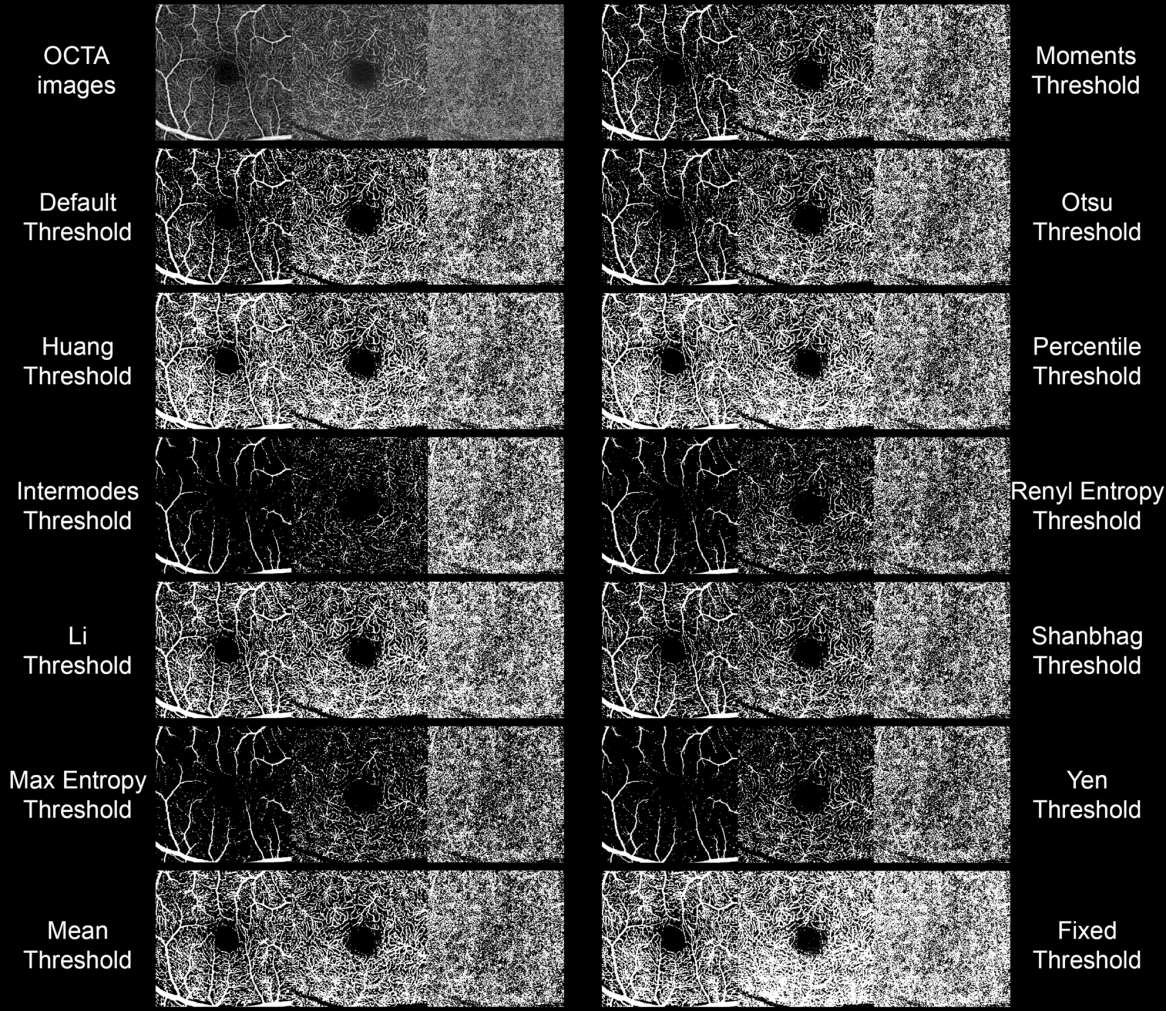
Binarized OCTA reconstructions obtained through all the included binarization techniques.

The results of all binarization thresholds used are shown in Fig. 2. The Quality Score values are reported in Table 2. The highest scores were reached by Fixed, Percentile and Huang thresholds both for SCP and DCP, and by Fixed and Percentile thresholds for CC. Fixed and Percentile thresholds resulted not statistically different for SCP and DCP (both p > 0.05), whereas Fixed threshold reached significantly higher score than Percentile threshold for CC (p < 0.05). Intermodes, MaxEntropy, RenylEntropy and Yen thresholding turned out to obtain the lowest scores.

**Table 2:** Quality score of binarized images. The two independent graders attributed to each binarized image a score ranging from 0 (< 20% of overlapping), 1 (< 80% of overlapping) and 2 (> 80% of overlapping).

Quality score			
Threshold	SCP	DCP	CC
Default	1.13 ± 0.35	1.17 ± 0.38	1.40 ± 0.50
Huang	1.73 ± 0.45	1.63 ± 0.49	1.60 ± 0.50
Intermodes	0 ± 0	0 ± 0	1.3 ± 0.47
Li	1.67 ± 0.48	1.50 ± 0.51	1.50 ± 0.51
MaxEntropy	0 ± 0	0.37 ± 0.49	1.20 ± 0.41
Mean	1.67 ± 0.47	1.57 ± 0.50	1.53 ± 0.51
Moments	1.20 ± 0.41	1.20 ± 0.41	1.37 ± 0.49
Otsu	1.40 ± 0.49	1.53 ± 0.51	1.53 ± 0.51
Percentile	1.80 ± 0.41	1.77 ± 0.43	1.67 ± 0.48
RenylEntropy	0 ± 0	0.13 ± 0.35	1.33 ± 0.48
Shanbhag	1.63 ± 0.49	1.60 ± 0.50	1.60 ± 0.50
Yen	0 ± 0	1.17 ± 0.38	1.30 ± 0.47
Fixed	1.93 ± 0.25	1.83 ± 0.38	1.97 ± 0.18

The quantitative evaluation of OCTA parameters is extensively reported in Table 3. Since Intermodes, MaxEntropy, RenylEntropy and Yen thresholding obtained the lowest Qualitative scores, we only reported the values, without considering these reliable. This was also proved by the fact that these thresholds overall provided the lowest VD and VT values, and the highest Vdisp values (p < 0.05). Fixed threshold provided the highest VD values for all the retinal plexa (all p < 0.05). Looking at VT and Vdisp measures, these resulted almost comparable among all the thresholding techniques (p > 0.05).

**Table 3:** Quantitative OCTA metrics measured for all binarized images.

	Default	Huang	Intermodes	Li	MaxEntropy	Mean	Moments	Otsu	Percentile	RenylEntropy	Shanbhag	Yen	Fixed
**Vessel density**													
SCP													
Mean	0.27	0.48	0.09	0.40	0.09	0.41	0.23	0.26	0.50	0.09	0.32	0.08	0.48
STD	0.04	0.01	0.03	0.02	0.01	0.01	0.03	0.04	0.01	0.01	0.03	0.01	0.03
DCP													
Mean	0.34	0.45	0.66	0.42	0.14	0.43	0.31	0.34	0.50	0.17	0.32	0.17	0.56
STD	0.02	0.01	0.43	0.02	0.01	0.01	0.02	0.02	0.01	0.01	0.03	0.01	0.01
CC													
Mean	0.50	0.50	0.26	0.54	0.38	0.49	0.47	0.49	0.50	0.39	0.49	0.41	0.68
STD	0.01	0.01	0.28	0.01	0.04	0.01	0.01	0.01	0.01	0.03	0.01	0.04	0.01
**Vessel tortuosity**													
SCP													
Mean	8.81	9.60	6.81	9.35	7.18	9.79	8.58	8.96	9.49	7.29	9.35	7.24	9.66
STD	0.52	0.20	0.28	0.22	0.26	0.21	0.21	0.21	0.17	0.23	0.30	0.24	0.36
DCP													
Mean	8.47	8.54	6.16	8.47	6.37	8.50	8.17	8.47	8.61	6.37	8.52	6.37	8.80
STD	0.39	0.18	0.26	0.13	0.22	0.16	0.26	0.25	0.31	0.24	0.25	0.23	0.20
**Vessel dispersion**													
SCP													
Mean	10.44	9.46	14.48	9.39	16.43	10.29	10.85	10.61	9.75	16.06	9.77	16.28	10.36
STD	6.24	4.30	13.43	4.42	12.36	3.91	8.31	6.35	3.52	12.07	5.63	9.20	4.92
DCP													
Mean	14.20	12.00	19.81	13.06	16.16	11.14	15.27	13.93	11.92	13.30	13.20	12.88	12.67
STD	5.64	5.18	8.68	6.45	6.26	4.17	5.06	4.78	3.55	7.56	5.30	8.01	4.73

**Figure 3:**
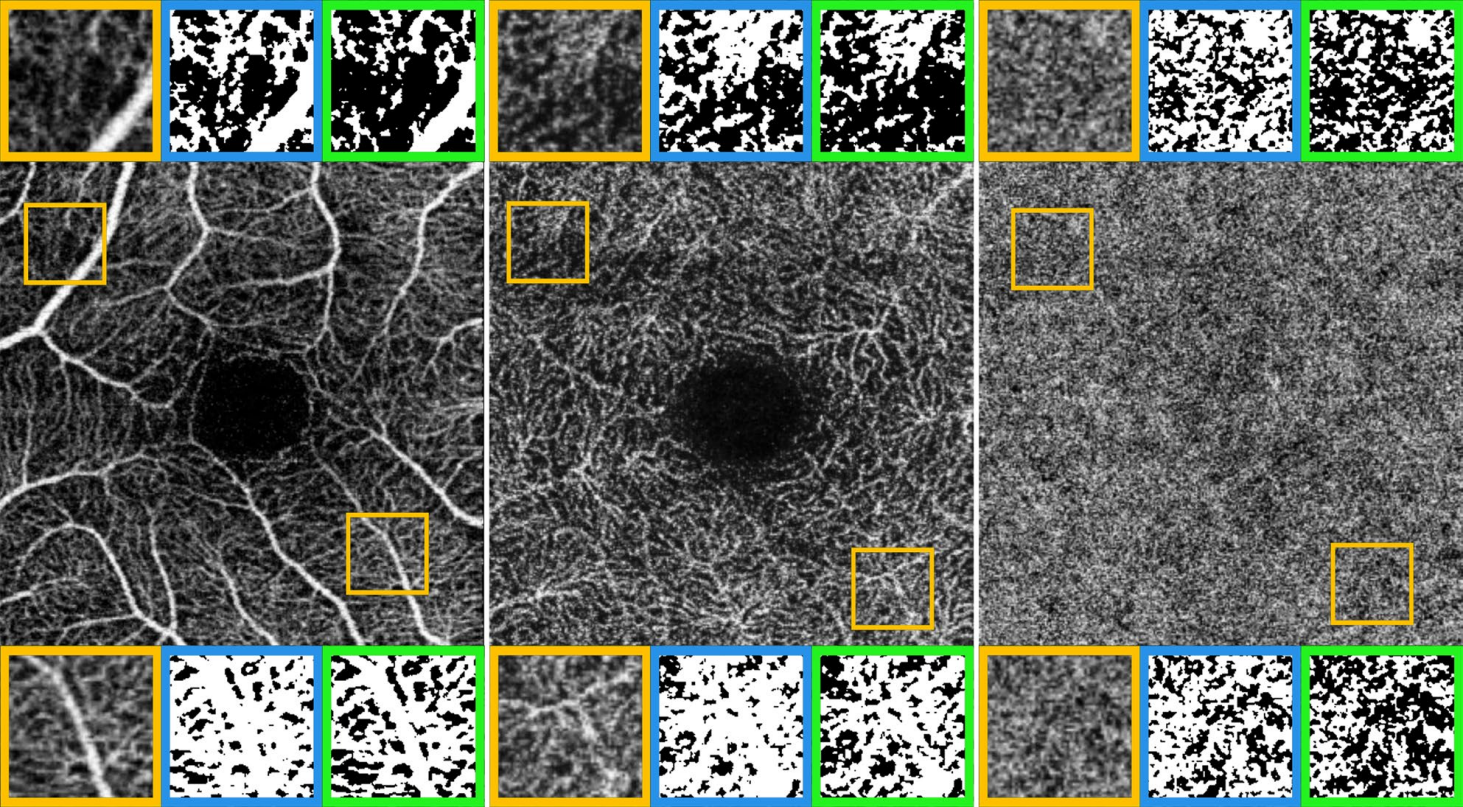
Fixed and Mean thresholding effects on OCTA images binarization. SCP, DCP and CC OCTA reconstructions are respectively shown in left, central and right images. Two detailed magnifications are taken in temporal and nasal sectors (orange squares). For all the retinal plexa, Fixed threshold (blue square) turned out to provide a more likely binarization result, compared to Mean threshold (green square) resulting in a visually evident underestimation.

**Figure 4:**
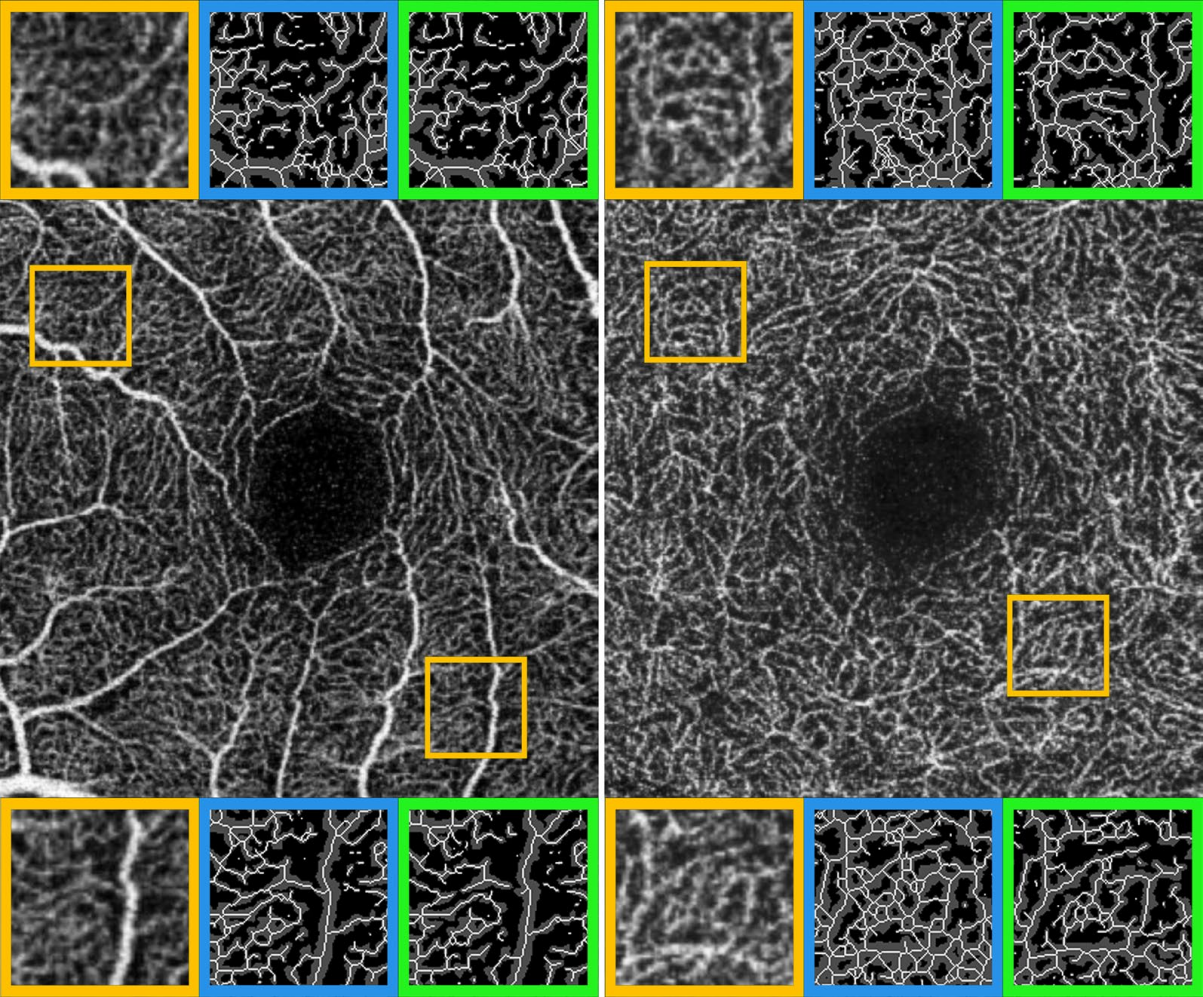
Fixed and Mean thresholding effects on OCTA images skeletonization. SCP and DCP OCTA reconstructions are respectively shown in left and right images. Two detailed magnifications are taken in temporal and nasal sectors (orange squares). Since the step preceding skeletonization is image binarization, both for SCP and DCP plexa, it is possible to observe also in this case an underestimation of retinal capillaries, provided by Mean threshold (green square) compared to Fixed threshold (blue square).

Details on the effects of Fixed and Mean thresholds on binarization and skeletonization processes are shown respectively in Figs. 3 and 4.

**Figure 5:**
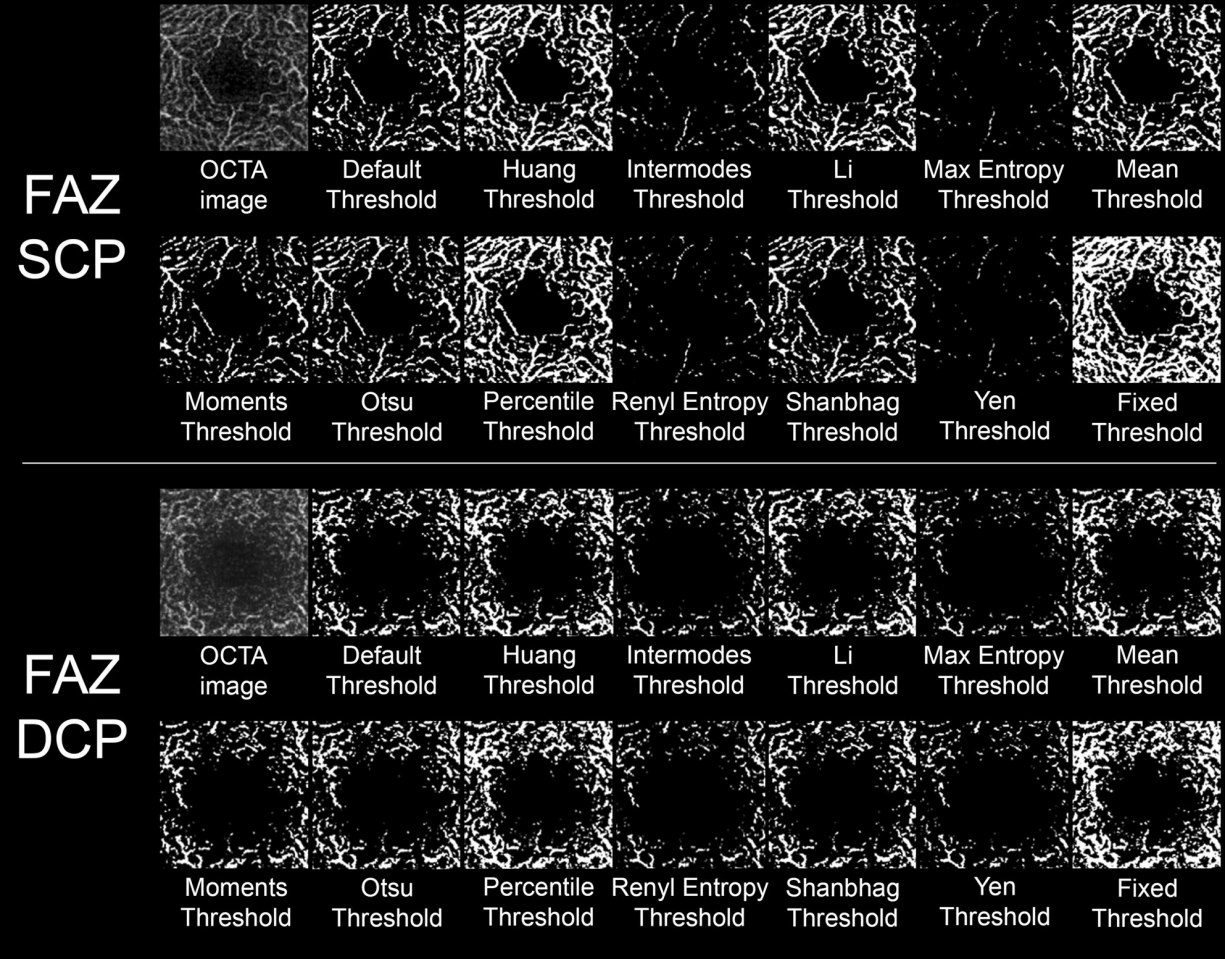
Binarized FAZ reconstructions obtained through all the included binarization techniques.

The results of all binarization techniques on FAZ detection are shown in Fig. 5.

**Figure 6:**
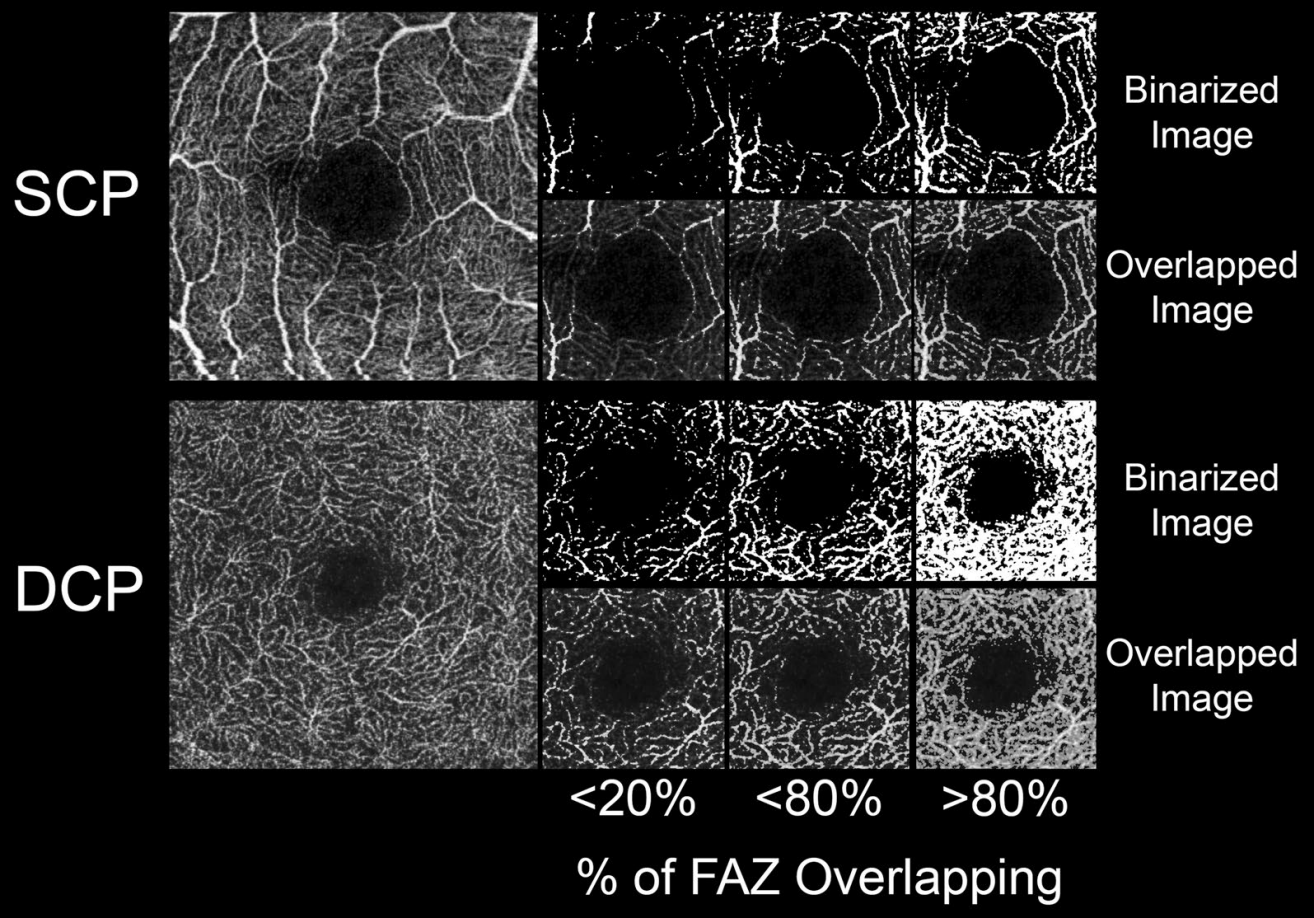
Examples of FAZ overlapping. The three FAZ scores (< 20% of overlapping, < 80% of overlapping and > 80% of overlapping) are respectively shown.

In this case, as described in the “Materials and methods” section, the two expert graders attributed a score to the binarized images, accordingly to the accuracy of the overlapping between the binarized FAZ and the original OCTA reconstruction, as follow: < 20% of overlapping, < 80% of overlapping and > 80% of overlapping (Fig. 6). The FAZ scores results are reported in Table 4. Fixed threshold turned out to be the most reliable in terms of FAZ overlapping, both for SCP and DCP (p < 0.05). Intermodes, MaxEntropy, RenylEntropy and Yen thresholds showed the worst percentages of overlapping, turning out to be exclusively characterized by eyes with 0% of FAZ overlapping (p < 0.05). Percentile and Huang thresholds showed significantly higher FAZ overlapping results with respect to the other thresholds, excluding Fixed thresholds (p < 0.05).

**Table 4:**
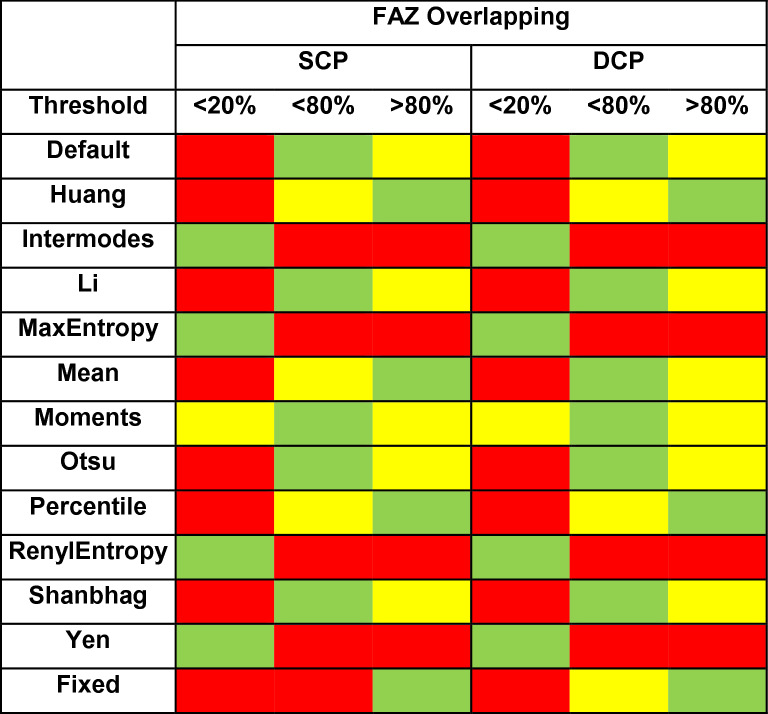
Colorimetric score of FAZ overlapping between binarized FAZ and original OCTA reconstruction. We stratified this overlapping in < 20%, < 80% and > 80%, both for SCP and DCP. Colors indicate the percentages of eyes, namely 0% (red), > 20% (yellow) and > 60% (green).

## Discussion

In the present study, we evaluated the results provided by thirteen different binarization thresholds. Our data showed statistically significant superiority of Fixed threshold, established at 65 for SCP and DCP, and 85 for CC, with respect to all the other thresholds. This was true for VD metric, calculated for all the vascular plexa, and for the detection of FAZ in SCP and DCP. On the other side, the VT and Vdisp values calculated after Fixed images’ thresholding turned out to be similar to the other thresholds. Intermodes, MaxEntropy, RenylEntropy and Yen thresholds resulted significantly less reliable for all the experiments performed. Huang, Li, Mean and Percentile thresholds showed highly reliable results, both looking at OCTA quantitative metrics and FAZ detection.

Image binarization is a crucial step for quantitative post-processing OCTA analyses. Although largely used in research context, a consensus regarding the choice of the best binarization threshold is still lacking. To the best of our knowledge, the impact of binarization threshold was evaluated considering only VD OCTA metric, turning out to be significantly affected by the choice of the thresholding technique^21,22^. Furthermore, our data highlighted that the choice of the binarization technique may have an influence also on the non-tubular structure of the CC, as reported by the significantly different VD values found among each binarized CC reconstruction. This was true also for FAZ detection^23^, for whom the automatic identification of the FAZ borders would be extremely useful to perform FAZ area analyses on large datasets^24^.

Resuming the results provided by previous studies, Mean threshold turned out to the most used binarization technique. In the present study, this threshold showed good results, both in terms of percentage of overlapping and quantitative OCTA metrics, although it was comparable with Huang, Li and Percentile thresholds. It is worth of notice that the employment of a Fixed threshold was able to provide significantly better results. This threshold was established by two independent expert graders, and differed between SCP/DCP and CC. Previous findings showed the superiority of automatic approaches with respect to manual ones^25^. From this point of view, we are aware that the usage of a Fixed threshold can be influenced by the experience of the graders, thus making possible to provide highly variable results. In the present study, the agreement between the graders was remarkably high, thus suggesting a potential role of a Fixed thresholding approach.

The employment of a Fixed threshold highlighted an overall underestimation of retinal capillaries quantification provided by other thresholds (Mean threshold used as reference), which was evident looking at VD values. On the other side, VT and Vdisp seemed less affected by the choice of the binarization threshold. A possible explanation is that, whereas VD represents an overall calculation of absolute image intensity, VT and Vdisp are focused on the geometric properties of the retinal capillaries. Hence, the underestimation of lower reflectivity signal might lead to significantly lower VD values, however having less influence on the properties of the skeletonized images.

Overall considering all the present findings and the technical aspects of each binarization techniques, looking at the automatic thresholding techniques, it appears that the winning approaches are those which somehow comprise a cross-check with the original image. Indeed, Mean thresholding approach, merely based on the distribution of the grayscale values of each image, calculates a “personalized” threshold value for each image, thus accurately preserving the morphological distribution of retinal capillaries. On the other side, the reason below the success of Huang, Li and Percentile thresholding techniques might lie in the fact that all these techniques include different approaches to check the distance between the grayscale original image and its binarized version^26^. On the other side, the lack of similar variance values distribution, the failure in adopting entropy maximization techniques and other possible reasons requiring further investigations might justify the unsuccessful results of the other tested thresholding approaches^26^.

We are aware that our study is potentially affected by possible limitations. A strong point of our investigation is the inclusion of several thresholds, with respect to other papers testing fewer binarization techniques. Moreover, compared to previous studies, we included remarkably higher number of eyes and quantitative OCTA metrics, although we acknowledge that the reliability of our findings would have benefit from the inclusion of higher number of eyes. A major limitation of the study is the lack of an objective reference to verify each binarization technique. In our paper, we compared our findings with “Mean” threshold, which is the most used binarization techniques and we also used the percentage of overlapping with the original OCTA reconstruction. However, we are aware that the use of histologic confirmation or the adoption of a properly built phantom would have improved the accuracy of our investigation. From this point of view, future studies should be conducted employing these kinds of references. Another weak point is the absence of tests including different OCTA devices, as well as the absence of multiple testing of the same device in order to assess intra-device reproducibility. From this point of view, we are encouraged by previous evidence reporting high reliability and reproducibility disclosed by Topcon Triton device^27^. Furthermore, several other thresholds techniques exist, which might potentially provide useful data. For this reason, further studies should assess the impact of thresholding techniques on different OCTA devices, including higher number of binarization thresholds. In addition, our FAZ analyses were mainly focused on the accuracy of FAZ detection after binarization process, without considering FAZ area measurement. This choice was done because of several thresholding techniques resulted poorly reliable in detecting FAZ, thus irremediably compromising FAZ area estimation. On the other side, the percentage of overlapping may be considered a reliable measure of output reliability. Furthermore, we included only high-quality data, poorly affected by artifacts, and our Fixed threshold was established exclusively on these healthy data. Future studies should be conducted including also images affected by different artifacts, together with eyes affected by retinal diseases.

In conclusion, our study reported Huang, Li, Mean and Percentile thresholds as highly reliable binarization techniques to be employed in OCTA quantitative metrics calculation and in FAZ detection. Although possibly influenced by graders’ experience, Fixed threshold turned out to be highly efficient and poorly underestimating VD measurement.
